# Fatty Acids are Key in 4-Hydroxy-2-Nonenal-Mediated Activation of Uncoupling Proteins 1 and 2

**DOI:** 10.1371/journal.pone.0077786

**Published:** 2013-10-28

**Authors:** Elena A. Malingriaux, Anne Rupprecht, Lars Gille, Olga Jovanovic, Petr Jezek, Martin Jaburek, Elena E. Pohl

**Affiliations:** 1 Institute of Physiology, Pathophysiology and Biophysics, University of Veterinary Medicine, Vienna, Austria; 2 Institute of Pharmacology and Toxicology, University of Veterinary Medicine, Vienna, Austria; 3 Institute of Physiology, Academy of Sciences of the Czech Republic, Prague, Czech Republic; University of Quebect at Trois-Rivieres, Canada

## Abstract

The production of reactive oxygen species (ROS) in mitochondria is very sensitive to the proton motive force and may be decreased by mild uncoupling, mediated e.g. by mitochondrial uncoupling proteins (UCPs). UCPs were conversely hypothesized to be activated by ROS. Conclusions from experiments studying the reactive product of lipid peroxidation 4-hydroxy-2-nonenal (HNE) in isolated mitochondria and UCP knock-out mice are highly controversial. Here we investigated the molecular mechanism of HNE action by evaluating the separate contributions of lipid and protein phases of the membrane and by comparing UCP1 and UCP2, which were reconstituted in planar lipid bilayers. We demonstrated that aldehyde does not directly activate either UCP1 or UCP2. However, HNE strongly potentiated the membrane conductance increase (G_m_) mediated by different long-chain fatty acids in UCP-containing and in UCP-free membranes and this suggest the involvement of both lipid-mediated and protein-mediated mechanisms with FA playing the central role. G_m_ increase was concentration-dependent and exhibited a typical saturation kinetic with the binding constant 0.3 mM. By using Electron Paramagnetic Resonance, membrane fluidity change could be excluded as a cause for the HNE-mediated increase in the presence of FA. The impact of the HNE binding to definite positively charged UCP amino acid residues is discussed as a possible protein-mediated mechanism of the UCP activation.

## Introduction

Free radicals and other reactive oxygen species (ROS) have been implicated in aetiology of numerous pathological states such as neurodegenerative disease, diabetes, obesity, cardiovascular disease, etc. [1], [2]. Diminishing mitochondrial ROS production is therefore an important therapeutic strategy in the treatment of diseases in which ROS are thought to be involved. Mitochondria produce a substantial amount of superoxide anion (O_2_^-^⋅), which is able to give rise to other ROS and lipid peroxidation products. Among them is 4-hydroxy-2-nonenal (HNE), which is formed during peroxidation of ω-6 PUFA side chains (linoleic,γ-linolenic, and arachidonic acid). HNE was shown to modify intracellular components, such as lipids, DNA and proteins [3], [4]. The biological effects of HNE on cells were reported to strongly depend on its concentration [5], [6]. HNE in a concentration of 100 µM and above causes unspecific cytotoxic effects. Concentrations in the range of 30 to 50 µM, used in the experiments with mitochondria, can inhibit DNA and protein synthesis, stimulate phospholipase A2 and inhibit c-myc expression, which is essential for mammalian cell proliferation. Significant protein conformational changes have already been observed at a physiologically relevant concentration of µM HNE (for review, see [6]).

According to the “mild uncoupling” hypothesis [7], a regulated H^+^ leak may lead to the attenuation of mitochondrial ROS production, as confirmed for superoxide formation at both Complex III [8] and Complex I sites [9]. Mitochondrial uncoupling proteins (UCPs) were proposed to be a part of a self-regulating system, where ROS and lipid peroxidation products activate UCP that leads to the proton motive force and membrane potential (ΔΨ_m_) decrease, followed by a decline of ROS generation [10]–[12]. Although such negative feedback seems to be an attractive hypothesis, it is highly controversial [13]–[15].

UCP1 activation by HNE was first demonstrated by Echtay et al. [16] in isolated rat mitochondria and supported by data obtained from plant mitochondria [17]. It was proposed that the aldehyde is able to convert UCPs into active protein transporters by their covalent modification [18]. In contrast, using UCP1 knock-out mice, Shabalina et al. demonstrated that HNE “neither (re)activates purine nucleotide-inhibited UCP1, nor induces additional activation of innately active UCP1” [13]. Comparing the re-activating ability of aldehyde and corresponding FAs (2-nonenoic and nonanoic acid) on UCP1 in the presence of GDP they showed that the presence of carboxyl groups is absolutely required [13]. Interestingly, this ability was independent on the degree of FA saturation. It contradicts our results, which demonstrated a clear correlation of UCP activity and the number of double bonds [19], [20].

No protein-related effect was found for superoxide anion radicals [14] that were previously reported to activate UCP1, UCP2 and UCP3 [21]. However, Parker et al. [22], [23] suggested that high ΔΨ_m_ is required for the activation of UCP-mediated uncoupling by HNE. The discussion was recently extended to the uncoupling protein of unicellular eukaryotes. Woyda-Ploszczyca and Jarmuszkiewicz demonstrated that HNE activates UCP in *Acanthamoeba castellanii* mitochondria during non-phosphorylating respiration [24].

In order (i) to test whether the reactive aldehyde 4-hydroxy-2-nonenal can directly activate UCP1, (ii) to evaluate the discrepancies obtained in experiments with mitochondria and (iii) to gain insight into the mechanism of this process, we now use the well-defined system of the artificial lipid bilayers [25]. The total membrane conductance of lipid membranes with reconstituted, highly purified protein is compared in the presence of HNE, fatty acids or both. The role of high potentials and the degree of FA saturation is investigated. Finally, we discuss the mechanism underlying the increase of proton leakage as seen in the presence of UCP, HNE and FAs.

## Materials and Methods

### mUCP1 and hUCP2 expression, extraction, purification, and reconstitution into liposomes

The production of murine uncoupling protein 1 (mUCP1) is reported in our previous work [20]. The same batch of inclusion bodies was used for the protein reconstitution. Human uncoupling protein 2 (hUCP2) was produced in bacterial strain BL21 (Novagen, Germany) as described previously [19]. Inclusion bodies (IB) containing UCP1 or UCP2 were purified, solubilized and incorporated into liposomes according to the procedures previously established [19], [20], [26]. Aggregated proteins were eliminated by centrifugation of the dialysate at 14000 g for 10 min. In order to eliminate the incorrectly folded protein, the supernatant was filled into a column containing 1 g hydroxyapatite (Bio-Rad, Germany) [27], [28]. To eliminate non-ionic detergent, the sample was incubated with Bio-Beads SM-2 (Bio-Rad, Germany) [29]. The total protein content in proteoliposomes was measured by Micro BCA Protein Assay (Perbio Science, Germany) according to the manufactureŕs instructions. The purity of the proteoliposomes was controlled by SDS-PAGE as described in [30] and subsequent silver staining was carried out according to [31]. The correct folding was assessed by the conductance measurements in the presence of protein activators and inhibitors as described in [25].

To investigate the effect of HNE the proteoliposomes were subsequently incubated with different concentrations of HNE for 30 min at 37°C. Proteoliposomes prepared in this way were used in EPR, electrophysiological and Western Blot experiments.

### Formation of planar membranes and measurements of membrane electrical parameters

Planar lipid bilayers were formed on the tip of disposable plastic pipettes as described previously [25]. A lipid extract from E. coli was chosen for the formation of bilayer membranes, because of its similarity to the mitochondrial inner membrane lipid (phosphatidylethanolamine, phosphatidylglycerol and cardiolipin; 67.0∶23.2∶9.8 w% respectively) and because our previous results indicated that UCP properly folds in this lipid. However, in contrast to the composition of eukaryotic lipids, the fatty acid residues of phospholipids and cardiolipins of E.coli do not contain polyunsaturated fatty acids. The typical profile of an E. coli fatty acid methyl ester fraction analyzed by GC-MS [32] was identified as methyl hexadecanoate (36%), methyl (Z)-11-octadecenoate (28%), methyl (Z)-9-hexadecenoate (2%), methyl octadecanoate (1%), methyl tetradecanoate (1%) and two cyclopropyl fatty acids, methyl 9,10-methanohexadecanoate 1 (20%) and methyl lactobacillate 2 (12%). The low amount of unsaturated fatty acid chains was advantageous for this study, because additional internal sources of oxidation were minimized.

Hexane, hexadecane, Na_2_SO_4_, K_2_SO_4_, MES, TRIS, TES, EGTA, arachidic (ArA, 20∶0), 9-cis,12-cis-Linoleic acid (LA, 18∶2, ω6) and arachidonic (AA, 20∶4, ω6) acids were purchased from Sigma Aldrich GmbH (Germany), 11(Z),14(Z), 17(Z)-eicosatrienoic acid (EA, 20∶3, ω3) came from Cayman Chemicals. FAs were added to the lipid phase prior to membrane formation. FA-containing liposomes were then mixed with proteoliposomes in required proportions. The concentration of FA in membranes was 15 mol%. An ethanol solution of HNE was obtained from Biozol GmbH (Eching, Germany).

Membrane formation and quality was monitored by capacitance measurements (0.74±0.06 µF/cm^2^). The capacitance depended neither on protein nor on fatty acid content. Current-voltage (I–V) characteristics of model membranes were measured with a patch-clamp amplifier (EPC 10, HEKA Elektronik Dr. Schulze GmbH, Germany). Membrane conductance G_m_ was determined at voltages within the range of 10–220 mV from a linear fit of the current-voltage relationship obtained at voltage intervals of 10–20 mV.

### Measurements of the membrane order parameter with Electron Paramagnetic Resonance (EPR) method

The 5-doxyl stearic acid spin label (5-DSA) was used for assessment of the order parameter S (inversely correlated with the membrane fluidity). A stock solution aliquot of the respective spin label in acetonitrile was placed in an Eppendorf reaction tube and the solvent was evaporated by a stream of nitrogen. Then the remaining thin film of the spin label was incubated with the liposomal suspensions for 10 min at 37°C under frequent agitation. The spin label/lipid ratio was 7.5 nmol/(mg lipid).

After cooling to room temperature (25°C), 50 µl of the suspension was aspirated into a 50 µl glass micropipette, which was subsequently placed in the resonator of the EPR instrument. The EPR measurements were performed using a Bruker EMX instrument equipped with a Flexline dielectric resonator ER4118X-MD5 (Bruker, Rheinstetten, Germany). The following parameters were used for measurements: microwave frequency 9.683 GHz, modulation frequency 100 kHz, modulation amplitude 1 G, time constant 0.163 sec, center field 3448.86 G, scan rate 35 G/min, sweep 100 G, sweep time 168 s, receiver gain 2×10^4^, resolution 2048, scans 3. The peak positions of the EPR spectra were analyzed by our internally-designed software. The positions of A_max_ and A_min_ were directly measured for all spin label molecules. The order parameter S was calculated from these line positions according to the equation 1 and 2 [33], [34].
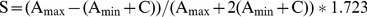
(1)

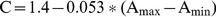
(2)


### Western blot analysis

As described previously [30], SDS-polyacrylamide gel electrophoresis was performed on 15% acrylamide gels loaded with 1 µl of proteoliposomes per lane. Proteins were transferred on nitrocellulose membranes. After incubation in block solution (1xTBS, 2% BSA, 0.5% Tween 20, 0.02% Thimerosal) overnight, membranes were incubated with HNE antibody (15 µg/ml, Abcam ab48506) in block solution two days at 4°C. Membranes were washed with buffer (1xTBS, 0.5% Tween 20) and subsequently incubated in horseradish peroxidase-linked anti-mouse antibody (GE Healthcare, UK) 1∶5000 in block solution for an hour. After washing, antibody-binding was visualized by ECL reagent (GE Healthcare, Germany) and measured with ChemiDoc-It-600 System (UVP, UK).

### Statistical analysis

Data are presented as mean ± SD. Statistical significance was determined using unpaired Student's t test. P values <0.05 were considered to be significant.

## Results

### Effect of HNE on the total membrane conductance (G_m_) in the absence and presence of UCP1

**Figure 1:**
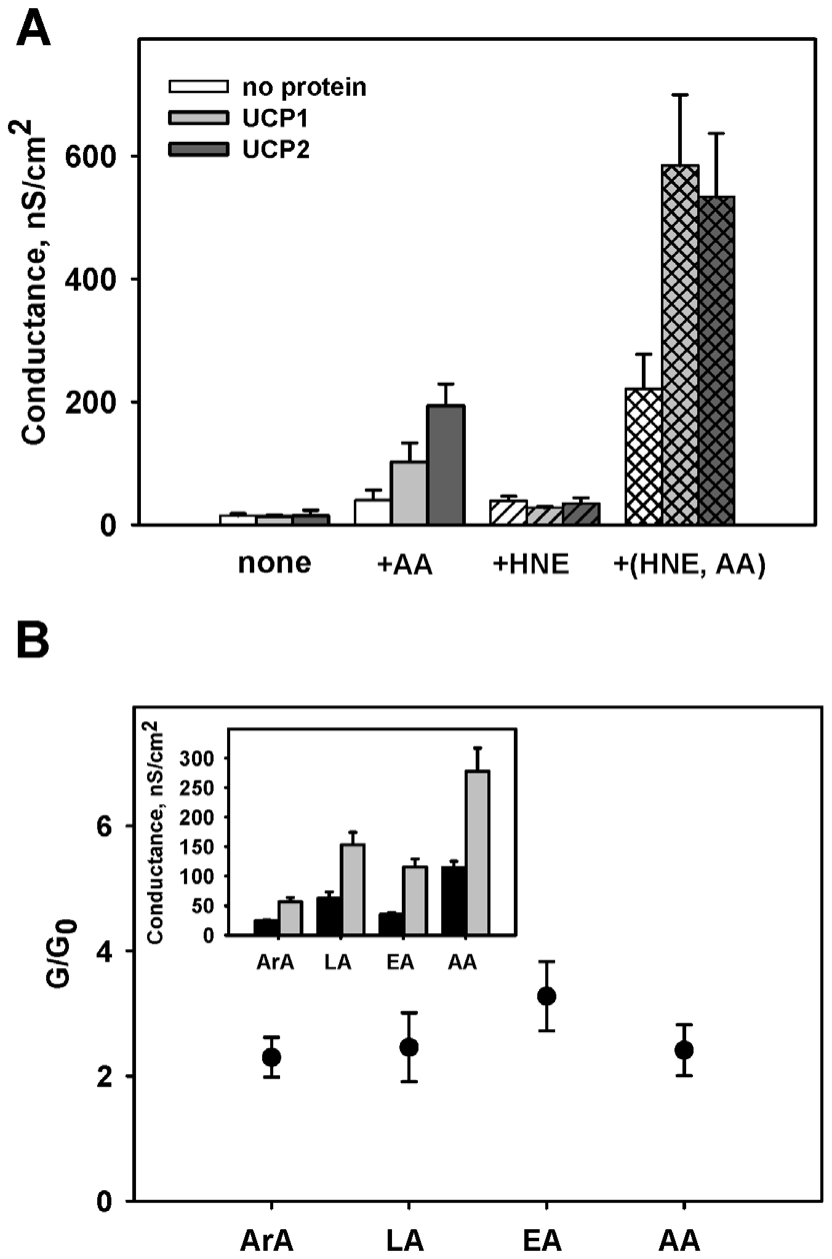
The effect of 4-hydroxy-2-nonenal on membrane conductance (G). A. Influence of HNE and/or arachidonic acid (AA) on G in the absence (white bars) and presence of UCP1 (grey bars) or UCP2 (dark grey bars). The concentrations of E. coli polar lipid, UCP1, UCP2 and HNE were 1 mg/ml, 5.5 µg/(mg of lipid, charge 9), 4.7 µg/(mg of lipid) and 0.86 mM respectively. AA was directly added at a concentration of 15 mol% to the lipid phase prior the membrane formation. The buffer solution contained 50 mM K_2_SO_4_, 25 mM TES 0.6 mM EGTA at pH 7.4 and T = 32°C. HNE was directly added to the buffer solution. Data points represent mean ± standard deviation from 3–5 independent experiments. B. Comparison of membrane conductance ratios in the presence (G) and absence (G_0_) of 0.64 mM HNE for arachidic (ArA, 20:0), linoleic (LA, 18:2, ω6), eicosatrienoic (EA, 20:3, ω3) and arachidonic acid (AA, 20:4, ω6). Inset. Membrane conductance in the presence (grey bars) and absence (black bars) of HNE. Membranes from E. polar lipid were reconstituted with 15 mol% FA and 7 µg UCP1/(mg of lipid) (charge 33). The buffer solution contained 50 mM Na_2_SO_4_, 10 mM Tris, 10 mM MES, 0.6 mM EGTA, at pH 7.4 and T = 32°C. Data represent mean ± standard deviation from at least 3 independent experiments.

In order to evaluate the direct action of HNE on uncoupling proteins, we reconstituted artificial bilayer membranes formed from E. coli lipid extract with UCP1. In agreement with our previous results [20], [25], the reconstitution of protein in the lipid membrane did not lead to the increase in *G_m_* if no further substitutes were added (Fig. 1, A, first bar set). In contrast, the reconstitution of UCP1 or UCP2 in the presence of arachidonic acid (AA, Fig. 1, A, second bar set and Fig. 1, B) led to the expected increase in the membrane conductance *G_m_*, similar to the data reported earlier [19]. The protein activation could be inhibited with ATP, as shown in the accompanying study using the same protein charge (Fig. 5 in [20]), ensuring proper protein folding. In order to evaluate its putative activating efficiency, HNE was added to lipid membranes without further additives (Fig. 1, A, third bar set, white bar) and to the membranes reconstituted with UCP1 or UCP2 (Fig. 1, A, third bar set, grey and black bars respectively). The comparison of protein-free membranes and membranes containing reconstituted proteins clearly demonstrated that no HNE-induced G_m_ increase occurred in the absence of FA.

In order to investigate the origin of proton conductance increase seen in mitochondria [16], we measured G_m_ in the presence of HNE and AA in both protein-free and protein-containing membranes. Fig. 1, A (fourth bar set) shows that in the presence of arachidonic acid G_m_ was increased after addition of HNE in both UCP-free (white bar), and UCP1(UCP2)-containing membranes (grey and black bars). Notably, the G_m_ increase in the presence of UCP, arachidonic acid and HNE was stronger than without protein. This excludes a simple additive effect and indicates a potentiation of the UCP1 and UCP2 activation by AA in the presence of HNE.

**Figure 2:**
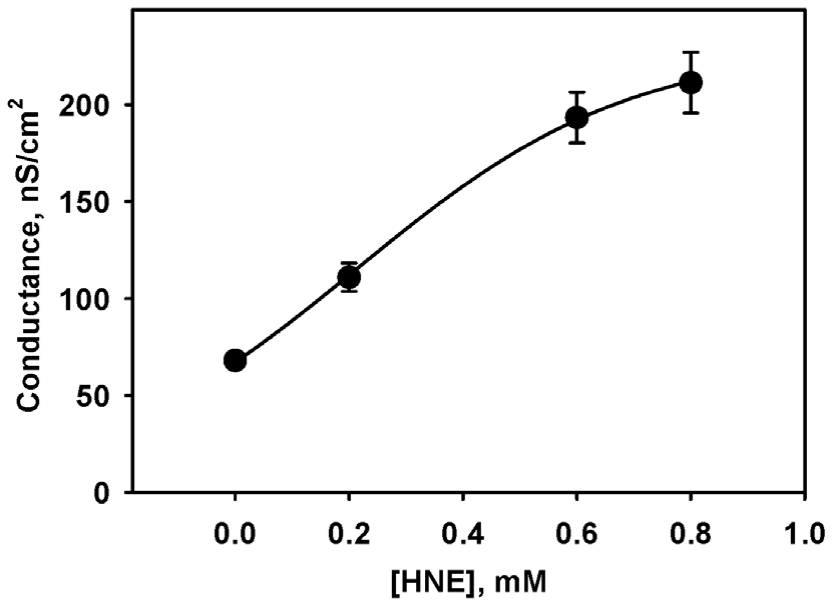
Dependence of UCP-mediated proton conductance on HNE concentration. Membranes from E. polar lipid were reconstituted with 15% arachidonic acid and 10.5 µg/(mg lipid) UCP1 (charge 19). Buffer solution contained 50 mM Na_2_SO_4_, 10 mM Tris, 10 mM MES, 0,6 mM EGTA, at pH 7.5 and T = 32°C. Data points represent mean ± standard deviation from at least 3 independent experiments. The data points were fitted following Michaelis-Menten kinetics as described in the text.

The potentiation of AA-mediated G_m_ increase was concentration-dependent and exhibited typical saturation kinetics at G_max_ = 303 mV (Fig. 2). The calculated binding constant corresponds to 0.3 mM. Only a slight G_m_ increase was seen at concentrations of 30-50 µM HNE used in the experiments of Echtay et al. [16], although the lipid to protein ratio in our experiments was higher.

To test whether the potentiating action of HNE is AA-specific we compared several FAs with different degrees of saturation, such as arachidic (ArA, G_m_ = 56.8±6.8), linoleic (LA, G_m_ = 153.3±21.3), eicosatrienoic (EA, G_m_ = 115.8±13.7). Arachidonic acid was measured (AA, G_m_ = 277.7±39.3) as a control for a new protein charge. The results revealed that although the absolute G_m_ is the highest in the presence by polyunsaturated FA and at the lowest after addition of saturated FA (Fig. 1, B, inset), the ratio G_m_/G_0_ is nearly constant for all tested FAs (Fig. 1, B).

### Effect of high membrane potential on the HNE-mediated membrane conductance in the absence and presence of UCP1

**Figure 3:**
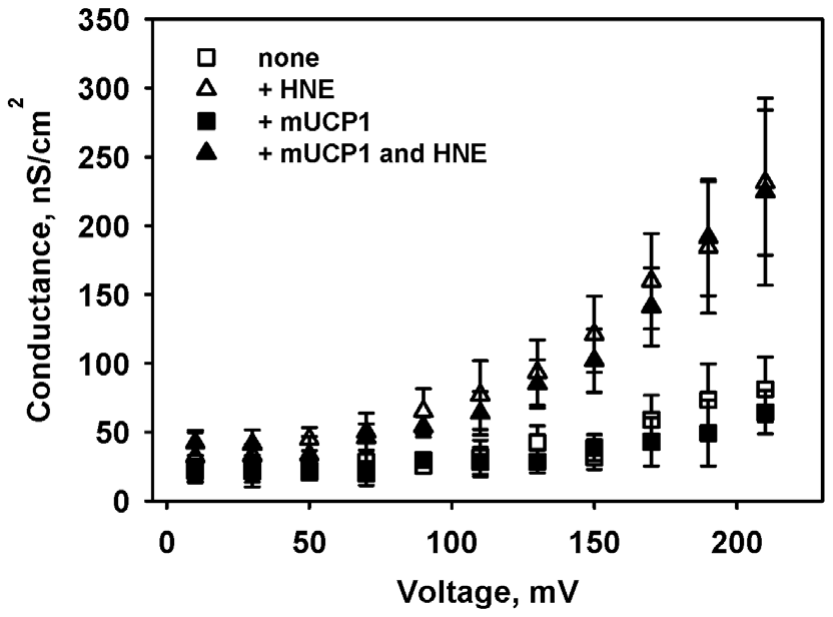
Effect of membrane potential on the HNE-mediated membrane conductance in the absence of AA. The concentration of membrane E. coli polar lipid, UCP1 and HNE were 1/ml, 5.5 µg/mg of lipid, and 0.86 mM. Buffer solution contained 50 mM K_2_SO_4_, 25 mM TES, 0.6 mM EGTA at pH 7.4 and T = 32°C. Data points represent mean ± standard deviation from 3–5 independent experiments.

In the next experiment we tested the hypothesis that stimulation of mitochondrial proton conductance by HNE requires a high membrane potential [22]. For this purpose, the G_m_ of membranes reconstituted with UCP1 and protein-free membranes were compared at potentials of up to 220 mV in the presence and absence of HNE. Figure 3 demonstrates a strong increase in the membrane conductance after the addition of HNE (compare white squares and white triangles), indicating the potentiating effect of the transmembrane potential on the membrane conductance. However, the comparison of protein-free (Fig. 3, white triangles) and protein-reconstituted membranes (Fig. 3, black triangles) demonstrated that the activating effect of HNE on G_m_ is protein-independent in the absence of FAs, even if high transmembrane potential is applied.

### Impact of AA and HNE on order parameters of the lipid bilayer

**Figure 4:**
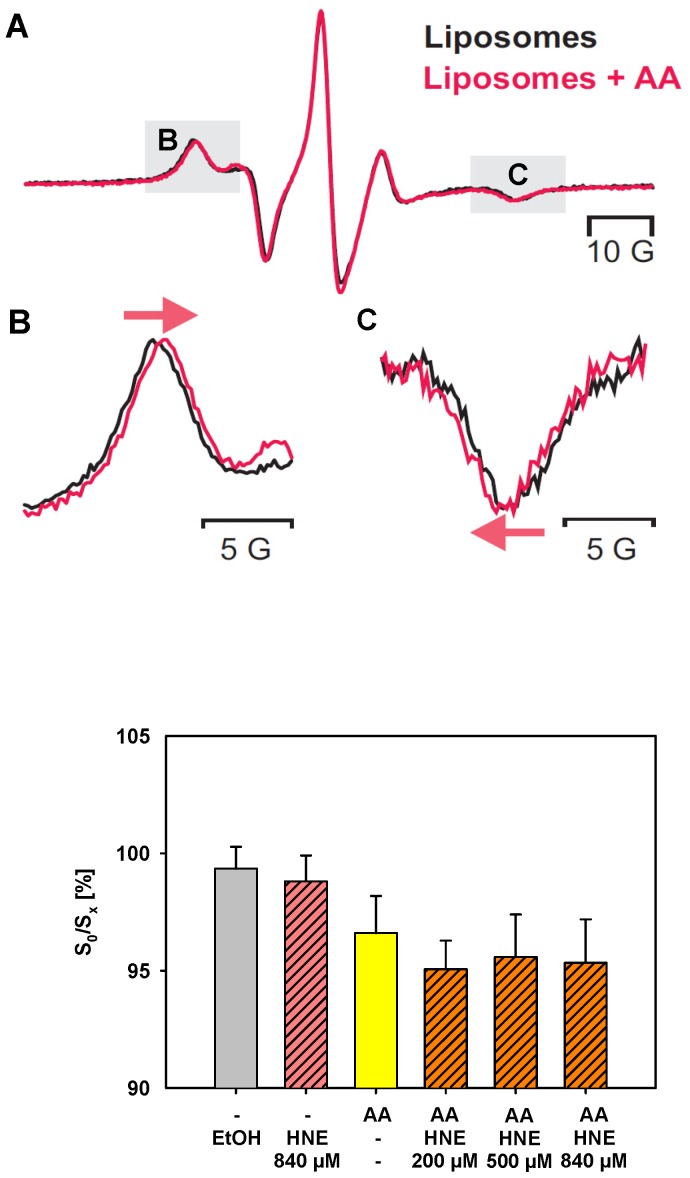
Alteration of order parameters in the presence of arachidonic acid (AA) and/or hydroxynonenal (HNE). Liposomes (5 mg/ml) were made from E.coli lipid. The concentration of the spin label 5-doxyl stearic acid (5-DSA) was 7.5 nmol/(mg lipid). Graph (A) shows a typical EPR spectrum of 5-DSA incorporated in liposomes in the absence and presence of AA. Details of the spectral shift in the low-field and the high-field line caused by AA are shown in (B) and (C), respectively. (D) The order parameter measured in control liposomes (S_0_) was set to 100 and all other order parameters (S_x_) were expressed in relation to this value. S_0_ and S_x_ were measured with or without additives (AA, HNE, Ethanol).

We used the electron paramagnetic resonance technique (EPR) (s. Methods, Fig. 4, A–C) to test whether arachidonic acid and/or HNE influence membrane fluidity by measuring the membrane order parameter S for liposomes without additives and for liposomes containing AA or HNE or both. Fig. 4, D shows that the addition of AA to the membrane decreased the membrane order parameter S, and therefore increased the membrane fluidity as previously hypothesized [19]. In contrast, HNE in maximal concentration did not lead to a significant increase in S. This was similar to the negative controls that contained ethanol (S_x_/S_0_ = 99.4%, Fig. 4, D) or saturated arachidic acid (S_x_/S_0_ = 98.3%). S_0_ and S_x_ stand for order parameters measured with or without additives (AA, HNE, and Ethanol). The addition of different concentrations of HNE also did not lead to any significant concentration-dependent increase in S in comparison to liposomes that were only reconstituted with AA (Fig. 4, D).

### Membrane conductance decrease in the presence of amino acid blockers

Most of HNE-mediated biological effects are attributed to its capacity to react with several amino acid residues of proteins, forming covalently modified biomolecules. The main HNE targets are cysteine, lysine and histidine [5], [35]–[38]. Because of 7 cysteine, 17 lysine and 3 histidine residues, UCP1 also represents a target for aldehyde binding.

To test whether the binding of amino acid blocking substances affects the observed HNE-mediated G potentiation in the presence of FAs, we used N-ethylmaleimide (NEM), sulfo-NHS-acetate (NHS) or metyl-4-nitrobenzen-sulfonate (MNBS), which competitively bind to cysteine, lysine or histidine respectively [37]. For these experiments, proteoliposomes containing UCP1 (0.48 nM) were incubated with NEM (0.5 µM, 100-fold molar excess per Cys residue), NHS (1.3 µM, 100-fold molar excess per Lys residue) or/and MNBS (0.2 µM 100-fold molar excess per His residue) for 1 h at 37°C according to [37], [38]. As an unmodified control, UCP1 was incubated with an equal volume of water. The proteoliposomes were than subsequently incubated with 840 µM HNE for 30 min at 37°C.

**Figure 5:**
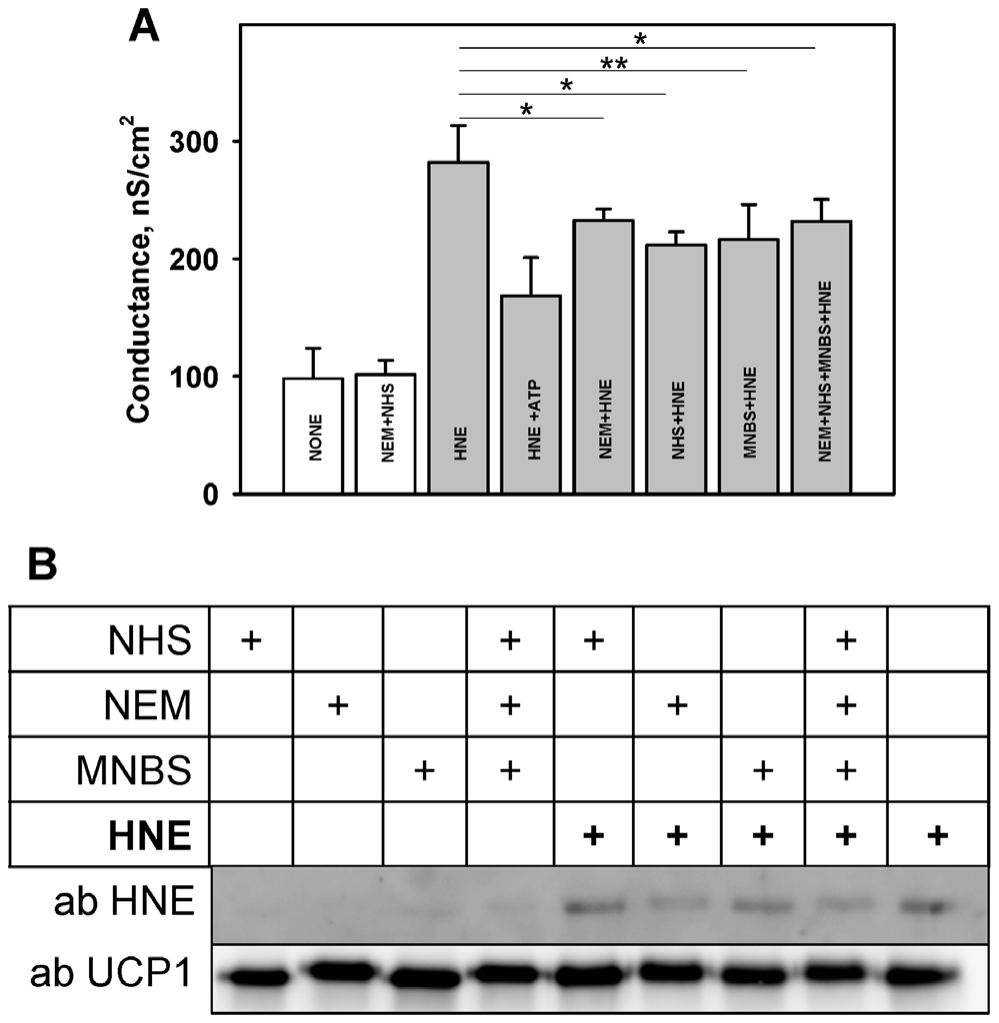
Analysis of HNE-mediated UCP1 modification. A. Membrane conductance decrease in the presence of Cys, Lys, and His blockers. Membranes were made from E. polar lipid (1mg/ml) and reconstituted with arachidonic acid (15 mol%) and UCP1 (charge 18, 11.9 µg/(mg of lipid). Bar 1 shows the conductance of the membrane without additives (control). NEM, NHS and MNBS in concentrations of 0.5 µM, 1.3 µM and 0.2 µM respectively were incubated with protein as described in Results. HNE was added in concentration 860 µM (grey bars). Buffer solution contained 50 mM Na_2_SO_4_, 10 mM Tris, 10 mM MES, 0.6 mM EGTA at pH 7.5. Data points represent mean ± standard deviation from 3–5 independent experiments. * p<0.05; ** p<0.01. B. Western blot analysis of the UCP1-containing proteoliposomes in the presence of different additives (arachidonic acid, AA; hydroxynonenal, HNE; NEM; NHS; MNBS). Only HNE-modified UCP1 is recognized by HNE antibody.

The addition of amino acid blockers (one by one or in different combinations) did not change the conductance of bilayer membranes if UCP and HNE were not present (Fig. 5, A, bar 1–2). In contrast, the HNE-mediated G_m_ (Fig. 5, A, bar 3) was significantly decreased in the presence of each (NHS or MNBS or NEM, Fig. 5, A, bars 5–7) and all substances (Fig. 5, A, bar 8), if the system contained both UCP1 and AA. Also, the combination of two substances (NHS+MNBS, or NHS+NEM or NEM+MNBS) gave similar results (data not shown).

Western blot analysis has shown that HNE-specific antibodies bind to proteoliposomes after their incubation with HNE (Fig. 5, B). However, even UCP1 pre-incubation with NHS, MNBS and/or NEM did not completely inhibit HNE binding, although the substances were added in excess (100 times more than the amount of corresponding amino acid residue). Incomplete inhibition of HNE binding can be partly explained by the fact that HNE was also reported to bind to arginines [39], however, with much lower affinity.

## Discussion

In the present work we evaluated the effect of the lipid peroxidation product, 4-hydroxy-2-nonenal (HNE), on two uncoupling proteins, UCP1 and UCP2. Difficulties in interpretation of results obtained in isolated mitochondria and UCP knock-out mice motivated us to use an experimental system essentially limited to two compounds (lipid and protein). Our data clearly show that HNE does not activate both UCP1 and UCP2 directly. These results are in line with data obtained for UCP1 using UCP1 knock-out mice [13], but contradict the results of other groups [16], [17], [24]. The latter have found HNE to be a strong activator of proton conductance mediated by mitochondrial uncoupling proteins UCP1, UCP2, UCP3, StUCP and AcUCP, using isolated rat, plant or amoeba mitochondria. Parker et al. [22] reported that “both endogenous HNE production and high membrane potential are required before mild uncoupling will be triggered to attenuate mitochondrial ROS production.” The direct application of high potentials (up to 220 mV, as observed in mitochondria), to the membranes, containing recombinant UCP revealed no direct alteration of uncoupling protein activity. Lack of direct HNE impact on UCP is not surprising if the “fatty acid cycling” hypothesis [40], [41] as mechanism for protein activation is presumed to be correct. This hypothesis postulates two steps in proton transport: (a) the flip-flop of protonated fatty acids along their interleaflet concentration gradient and (b) the subsequent backward transport of deprotonated (anionic) fatty acids by UCP. The proton transport in the presence of aldehydes can only be measured under special conditions, e.g. alcohol dehydrogenase and NADH [42], which are present in cells but were excluded in our model system. Our results support the data of Shabalina et al. [13] that UCP1 activation requires the presence of carboxyl groups.

Although no direct UCP activation by HNE was observed, it further enhanced the membrane conductance if added in the presence of different (saturated and unsaturated) FAs (Fig. 1, A and B). These data partly confirm the results obtained using mitochondria [43]. However, the authors showed that HNE had already increased proton conductance when added alone, palmitate synergistically enhanced this effect. The fact that we have not found the effect of HNE alone has led us to interpret our data as a potentiation of the FA-mediated membrane conductance increase by HNE rather than synergic action of both substances. In light of these data, we assume that the effect of HNE in mitochondria was due to the presence of small amounts of endogenous FA. The conductance of membranes reconstituted with AA in our experiments was increased in the concentration-dependent manner starting with approximately 50 µM (concentration used in [16]) up to 0.86 mM (measured in cells under conditions following oxidative stress [5], [44]).

Because of the pronounced lipophilic properties, it was previously proposed that HNE would preferentially affect (i) membrane lipids and/or (ii) membrane proteins. (i) HNE can alter the physical state of lipids, which in turn can directly affect the transport properties of membranes or influence the function of transmembrane proteins as described for high HNE concentrations [45]. Changes of lipid membrane properties, such as the alteration of membrane fluidity, loss of phospholipids asymmetry and chemical modification of lipids (for example, phosphatidylethanolamine, [46]) were suggested. Because an increase in the membrane conductance was observed in our experiments to a lesser extent even without protein, but always in the presence of FA [20], we initially considered that the alteration of the membrane fluidity might be the reason. EPR study showed that the presence of arachidonic acid decreases the membrane order parameter S, supporting our hypothesis that the increase in membrane fluidity after addition of unsaturated fatty acids leads to UCP activity increase [19]. In contrast, the addition of aldehyde in different concentrations clearly showed the absence of the HNE effect on the membrane order parameter. It rules out the hypothesis that the facilitation of FA flip-flop may be due to the alteration of membrane fluidity by HNE. Interestingly, our results differ from findings of Subramaniam et al. [45] who reported the membrane fluidity increase in synaptosomes after treatment with HNE. This difference could be due to the differences in membrane composition: synaptosomes contain proteins in addition to lipids; in contrast, pure lipid was used in our study. Another reason may be different measurement conditions. In experiments described in this study, all spin label molecules were located in the lipid phase (Fig. 4, A, no isotropic signal from 5-DSA in the aqueous phase), while in the study of Subramaniam et al. an excess of spin label over lipids was used, giving rise to an additional isotropic signal from 5-DSA (Subramaniam et al., Fig. 5, C). This suggests that the artificial liposomal system, used in this study provides more direct information about HNE/lipid interactions.

**Figure 6:**
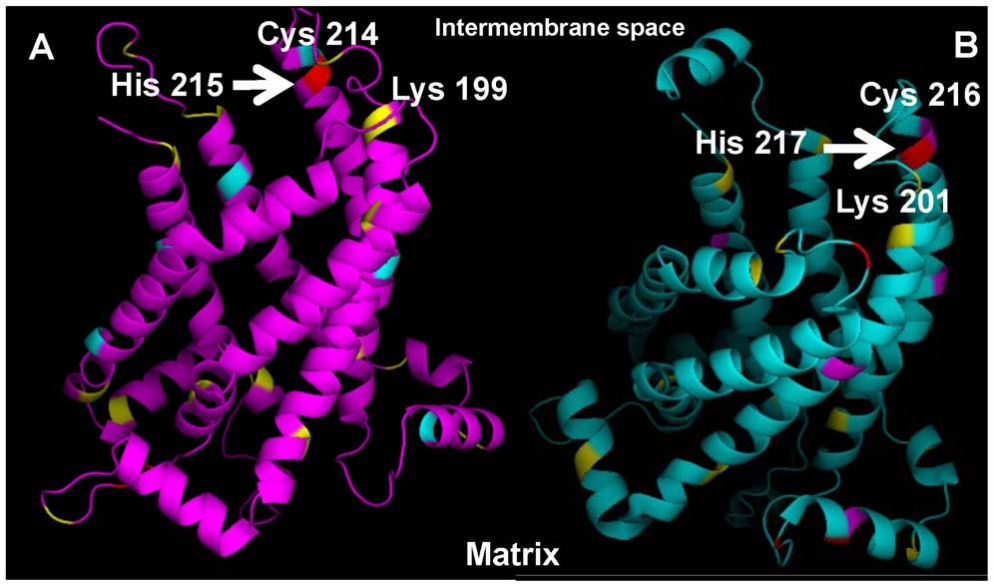
Position of cysteines (pink or mint), lysines (yellow) and histidines (red) in UCP1 (A) and UCP2 (B), visualized with PyMOL.

Although HNE did not influence membrane fluidity, the fact that membrane conductance increased without any protein, but in the presence of FA and HNE, indicated the participation of a lipid-mediated mechanism and FA's central role in it. Unfortunately, it is difficult to prove whether the modification of aminophosholipids (e.g. phosphatidylethanolamine) as proposed in [46] would influence the membrane conductance in our system, because the alteration of the membrane composition itself strongly influences G_m_. The incomplete inhibition of HNE-mediated G_m_ increase by NHS, MNBS and/or NEM (see (ii)) can be a further indication for two independent mechanisms underlying the effect of HNE.

(ii) The direct modification by HNE has already been reported for several proteins [10], [37], [47]. Saturation kinetics of the concentration dependence (Fig. 2) and HNE binding to the protein demonstrated by Western Blot analysis using anti-HNE antibody both support the idea that HNE may affect UCP by direct binding. Indeed, the competitive inhibition of HNE binding by several reagents known to bind to Cys, Lys and His residues reduces the increase in HNE-mediated G_m_. Interestingly, the maximal inhibition of each residue (Cys, Lys or His) or all residues together led to a similar decrease in membrane conductance, implying that the localization of HNE binding residue and not the amount of occupied binding sites is important. Moreover, despite the following order of binding potency Cys>>His>Lys [48], no difference in inhibition was determined in our experiments. To obtain some further clues about the inhibition mechanism, we visualized the HNE binding sites in both studied proteins, based on NMR-structure of UCP2 [49], using PyMOL Software (Fig. 6). Two His residues of UCP1 (His 146 and His 148, marked in red, Fig. 6, A) are located at the loop between transmembrane domain III and IV exposed to mitochondrial matrix and are not likely to contribute to the protein conformation change. The third His residue (His 215, marked in red, Fig. 6, A and B) is localized at the lipid-protein boundary facing the inter-membrane space and is a good candidate for the covalent binding of lipophilic HNE followed by putative protein modification. At the same location (at the bottom of the cytosolic vestibule) we find other putative residues for the HNE binding: Cys 214 (pink, Fig. 6, A), Lys 199 (yellow, Fig. 6, A). The identical degree of membrane conductance inhibition in the presence of NEM, NHS and MNBS implies that HNE binding to any molecules at this location may be sufficient for the initiation of the protein conformational changes. The comparison of UCP1 structure with that of UCP2 (Fig. 6, B) reveals the presence of similarly localized residues His 217, Cys 216, Lys 201, which would explain the similarity of HNE action on both UCP1 and UCP2.
